# Validity and Reproducibility of a Dietary Questionnaire for Consumption Frequencies of Foods during Pregnancy in the Born in Guangzhou Cohort Study (BIGCS)

**DOI:** 10.3390/nu8080454

**Published:** 2016-07-28

**Authors:** Ming-Yang Yuan, Jian-Rong He, Nian-Nian Chen, Jin-Hua Lu, Song-Ying Shen, Wan-Qing Xiao, Fang Hu, Hui-Yun Xiao, Yan-Yan Wu, Xiao-Yan Xia, Yu Liu, Lan Qiu, Ying-Fang Wu, Cui-Yue Hu, Hui-Min Xia, Xiu Qiu

**Affiliations:** 1 Division of Birth Cohort Study, Guangzhou Women and Children’s Medical Center, Guangzhou Medical University, 9 Junsui Road, Tianhe District, Guangzhou 510623, China; ymyeasy@126.com (M.-Y.Y.); Hjr0703@163.com (J.-R.H.); cnnfezx@163.com (N.-N.C.); lujinhua10@gmail.com (J.-H.L.); shsy_22@163.com (S.-Y.S.); xiaowanqing87@163.com (W.-Q.X.); hufang0351083@126.com (F.H.); xiaohuiyun1982@126.com (H.-Y.X.); kiky205@163.com (Y.-Y.W.); mountain_xia@163.com (X.-Y.X.); mzmxly81@163.com (Y.L.); mewmewprincess@hotmail.com (L.Q.); mywachy@163.com (Y.-F.W.); bighcy@163.com (C.-Y.H.); 2 Department of Neonatal Surgery, Guangzhou Women and Children’s Medical Center, Guangzhou Medical University, 9 Junsui Road, Tianhe District, Guangzhou 510623, China

**Keywords:** validation, dietary assessment, pregnant women, food frequency questionnaire, China

## Abstract

This study aimed to examine the reproducibility and validity of a new food frequency questionnaire (FFQ) used in a birth cohort study to estimate the usual consumption frequencies of foods during pregnancy. The reference measure was the average of three inconsecutive 24 h diet recalls (24 HR) administrated between two FFQs, and the reproducibility was measured by repeating the first FFQ (FFQ1) approximately eight weeks later (FFQ2). A total of 210 pregnant women from the Born in Guangzhou Cohort Study (BIGCS) with full data were included in the analysis. The Spearman’s correlation coefficients of FFQ1 and FFQ2 ranged from 0.33 to 0.71. The intraclass correlation coefficients of the two FFQs ranged from 0.22 to 0.71. The Spearman’s correlation coefficients of the 24 HR and FFQ2 ranged from 0.23 to 0.62. Cross-classification analysis showed 65.1% of participants were classified into same and contiguous quintiles, while only 3.2% were misclassified into the distant quintiles. Bland-Altman methods showed good agreement for most food groups across the range of frequencies between FFQ1 and FFQ2. Our findings indicated that the reproducibility and validity of the FFQ used in BIGCS for assessing the usual consumption frequencies of foods during pregnancy were acceptable.

## 1. Introduction

Dietary factors during pregnancy are very important for maternal health as well as for fetal growth [1,2]. Existing evidence has shown that an imbalanced maternal diet during pregnancy is associated with multiple adverse pregnancy outcomes, such as gestational diabetes mellitus (GDM), gestational hypertension, pre-eclampsia, fetal growth restriction, preterm birth, low birth weight, and macrosomia [1,2,3,4]. Assessment of foods intake during pregnancy can provide important information on dietary adequacy. The Born in Guangzhou Cohort Study (BIGCS) is an ongoing longitudinal study which would recruit 30,000 pregnant women by 2018 and follow-up their offspring until 18 years old [5]. The BIGCS aims to identify effects of environmental factors in early life on later health. Information on dietary intake, a crucial exposure, is collected during the middle of pregnancy (24 to 28 weeks) in the BIGCS. 

There are some challenges in the dietary survey among pregnant women. Eating habits might be changed after conception, after receiving the obstetrician’s professional counseling, or due to nausea or vomiting tending to resolve after the 1st trimester [6]. In addition, due to the rapid changes in maternal and fetal tissues and the increased basal metabolic rate during pregnant, extra energy intake for pregnant women is required, especially in the second and third trimester [7,8]. Most pregnant women may increase the amount of foods intake from early to later pregnancy. Therefore, it is difficult to estimate the dietary intake precisely by recalling a long period such as three months, six months or the whole period of pregnancy. 

In China, there have been several studies evaluating the reproducibility and validity of food frequency questionnaires (FFQs) used in pregnant women, but they were administrated in different areas including northern, central and western parts of China [9,10,11]. The dietary intake is culture specific, which can be observed differently among various demographic groups and subcultures even within a population from the same country [12]. As a vast and multi-ethnic country, dietary habits in China differ substantially between different regions and populations. So far there is no validation study of FFQ among pregnant women in the Cantonese area, a southern part of China where people have their own special culture and eating habits. In addition, materials used in Chinese dishes are really diverse and complex. In traditional Chinese culture, family members prefer to share their dishes with each other and pick up food in small chunks as they have meals. Thus, recalling the accurate amount of foods consumption during a long period might be difficult for Chinese subjects. Considering the time constraints of the interview and the challenges of recalling the food portion size, we designed a new dietary questionnaire (FFQ) for recalling the frequencies of foods intake in the previous week without collecting the portion size. The frequency component collected by the FFQ has been used to estimate the dietary patterns of pregnant women in the BIGCS studies [13,14].

The present study aimed to examine the validity and reproducibility of the FFQ for assessing the usual frequencies of foods consumption in Chinese pregnant women by comparing the frequencies of food items or groups between the FFQ and 24 h diet recalls (24 HR).

## 2. Materials and Methods 

### 2.1. Sample Recruitment and Study Design

**Figure 1:**
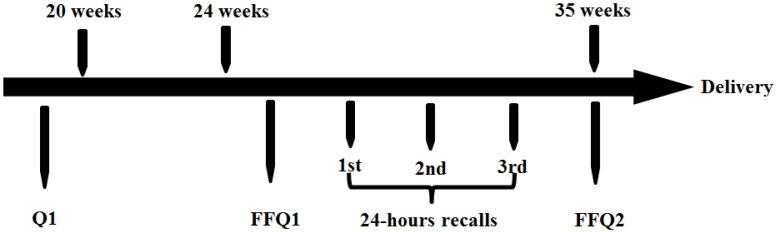
The design of the food frequencies questionnaire (FFQ) reproducibility and validation study among 210 pregnant Chinese women in Guangzhou, July 2014 to February 2015.

**Figure 2:**
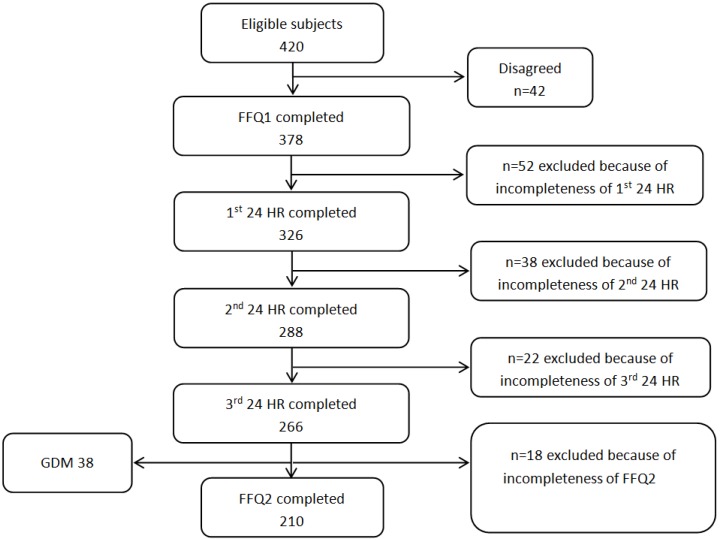
Flow diagram of sample selection. FFQ1: the first food frequency questionnaire; FFQ2: the second food frequency questionnaire (FFQ2); 24 HR: 24 h diet recalls; GDM: gestational diabetes mellitus.

The inclusion criteria of the BIGCS were: less than 20 weeks gestation, intended to have routine antenatal examinations in the Guangzhou Women and Children’s Medical Center (GWCMC) and resided within Guangzhou. A detailed questionnaire (Q1) about basic information such as sociodemographic characteristics, lifestyle, medical and obstetric history was required to be completed before 20 gestational weeks. The FFQ collecting the usual frequencies of foods intake in the previous week was administered between 24 and 28 weeks of gestation. The recruitment for this validation study was conducted from July 2014 to February 2015. A total of 420 women who agreed to participate in BIGCS and completed Q1 were eligible for this study and 378 of them agreed to attend the validity study. Subjects completed the first FFQ (FFQ1) at approximately 24–28 weeks gestation and the second FFQ (FFQ2) at around 32–35 weeks gestation. The inconsecutive three 24 HR, including two weekdays and one weekend, were collected on randomly selected days between the days on which the FFQ1 and FFQ2 administered. The study design was shown in Figure 1. Participants were included in the analysis if they completed both two FFQs and all three 24 HR. The women diagnosed with Gestational Diabetes Mellitus (GDM) were excluded due to the possible dietary changes after the diagnosis. Finally, 210 women with full data were included in this analysis (Figure 2). This study has been approved by the Institutional Ethics Committee of the Guangzhou Women and Children’s Medical Center.

### 2.2. Food Frequency Questionnaire

The FFQ, based on Dietary Guidelines for Chinese Residents [15] and the eating habits of Cantonese people, consisted of 64 food items. A total of 19 main food groups were constructed based on the FFQ, including red meats, poultry, eggs, fish, seafood, soybeans, other legumes, leaf vegetables, root vegetables, melon vegetables, mushrooms and fungus, seaweed, pickled vegetables, fruits, nuts, milk, cereals and grains, yogurt, and soup (Table S1). The food grouping was generally based on the similarity of nutrient profiles or culinary usage of the foods, mainly according to China Food Composition (second Edition) [16]. For each food item, participants were asked to report how many times they consumed the food over the past week (times/week). Both of the two FFQs (FFQ1 and FFQ2) have to be filled out by the pregnant women themselves in the obstetric clinic interviewing room. There were at least two trained interviewers in the interviewing room to help the participants if they had any questions about the FFQ. 

### 2.3. 24-h Diet Recalls (24 HR)

The repeated 24 HR were used as the reference method to validate the FFQ. In this study, three repeated 24 HR were used to collect the dietary information of pregnant women during the intervals of two FFQs. The inconsecutive three 24 HR included two weekdays and one weekend day and avoided the day when women had an illness (e.g., fever, diarrhea, or other gastrointestinal diseases). The trained interviewer made a phone call to the participants and asked them to recall the consumption frequencies in the previous 24 h of all foods, such as dishes, fruits, and snacks. The previous 24 h period was defined as the 24 consecutive hours between bedtime on day one and bedtime on the following day. For example, if the participant ate rice at breakfast, lunch and dinner, the frequency of rice consumption during previous 24 h is three. 

### 2.4. Statistical Analyses

All statistical analyses were performed using SPSS version 17.0 (SPSS Inc., Chicago, IL, USA). For the FFQ, daily frequencies were calculated as the total frequencies of each food consumed during the past seven days divided by seven. For the 24 HR, the daily frequencies were calculated as the total frequencies of each food recorded in the three 24 HR divided by three. The frequencies of food groups were calculated by adding the frequencies of each food items belonging to the same group. Data of frequencies of food groups were not normally distributed, thus the differences of frequencies between the two FFQs (FFQ1 vs. FFQ2) and between the two methods (24 HR vs. FFQ2) were tested by using Wilcoxon’s signed rank test. Spearman correlations were used to assess the reproducibility of the two FFQs and comparative validity between FFQ2 and average of three 24 HR. Intraclass correlation coefficients (ICCs) were calculated to evaluate the reproducibility of the two FFQs. ICCs are designed to assess consistency or conformity between two or more quantitative measurements where a higher value indicates lower within-subject variability in the response; they have been used previously to examine the agreement between repeated FFQs [17,18]. The Bland-Altman method was also used to examine the agreement between the two FFQs [19]. The mean differences between the two FFQs (FFQ1 and FFQ2) were plotted against the average frequencies of food items of the two FFQs. The 95% limit of agreement was calculated as the mean difference ± 1.96 SD [19]. 

The agreement of FFQ2 with the average of three 24 HR was also estimated by a quintile cross-classification analysis. According to the frequencies of food groups, the proportion of pregnant women classified into the same, same and adjacent, and distant quintiles was calculated. Misclassification into the distant quintile was defined as both misclassification from the first to the fifth quintile and from the fifth to the first quintile. In all analyses, statistical significance was set as *p* < 0.01. 

## 3. Results

### 3.1. Subject Characteristics

Table 1 shows the main characteristics of the validation study subjects (*n* = 210), non-participating validation study women (*n* = 210) as well as the BIGCS population (*n* = 10,165). There were no significant differences between participants and non-participants of validation study in education level, monthly income, pre-pregnancy body mass index (BMI) and previous parity. Compared with cohort population, the validation study group was better educated and included more primiparas (Table 1). 

### 3.2. Reproducibility

The median and mean daily consumption frequencies of the food groups from the two FFQs are shown in Table 2. In general, there was no significant difference in the frequencies of most food groups between the two FFQs (*p* > 0.05). There were slight differences in the frequencies of melon, vegetables and nuts between the two FFQs. The average Spearman’s correlation coefficient of the two FFQs was 0.45, ranging from 0.33 (seaweed) to 0.71 (soup). The ICCs for food groups ranged from 0.22 for pickled vegetables to 0.71 for soup with average of 0.42.

The Bland-Altman plot analysis graphs (Figure S1) show the agreement between the two FFQs. There is no dependency between the difference of the two FFQs and the average frequencies of food times from the two FFQs. Overall, the mean difference between the two FFQs was near zero (≤0.1) for the average consumption frequencies, except for melon vegetables. The limits of agreement of melon vegetables were −0.64 times/day to 0.93 times/day. 

### 3.3. Validity

Table 3 presents the median daily frequency assessed by FFQ2 and the three 24 HR. There were some food groups with a higher frequency in FFQ2, including root vegetables, melon vegetables, seafood, soybean, other legumes, yogurt, leafy vegetables, eggs, milk, and nuts. The frequencies of red meat, poultry, fish, mushrooms and fungus, fruits and soup were slightly higher in the 24 HR. The median Spearman correlation coefficient for the daily consumption frequencies estimated by the two methods was 0.34, ranging from 0.23 (pickled vegetables) to 0.62 (milk) (Table 3). 

Table 4 shows the results of the cross-classification analysis. The average percentage of classification into the same quintile, same or adjacent quintile, and distant quintile were 28.1%, 65.1% and 3.2%, respectively. The proportion of study subjects was over 60% categorized into the same or adjacent quintile for estimating the frequencies of food groups including seaweed, root vegetables, yogurt, cereals and grains, red meats, mushrooms and fungus, poultry, melon vegetables, soup, fish, nuts, fruits, eggs, seafood and milk. On average across all food groups, only 3.2% were misclassified in a distant quintile.

## 4. Discussion

A new FFQ was developed to estimate the usual consumption frequencies of foods during pregnancy in a population-based prospective birth cohort study (BIGCS) in Guangzhou of China. The range of correlation coefficients for most of the food groups, for both reproducibility and validity was between 0.3 and 0.7 in this study. In Willett’s report, the correlations between 0.3 to 0.7 in the epidemiology studies were in the acceptable to good range [21]. Therefore, the FFQ has an acceptable reproducibility and validity for assessing the usual consumption frequencies of foods during the pregnancy in BIGCS.

In the reproducibility study, The Bland-Altman analysis showed good agreement for most food groups across the range of consumption frequencies between the two FFQs (FFQ1 and FFQ2). The frequencies derived from the two FFQs were similar. The median correlation coefficient of the two FFQs was 0.45 (0.33–0.71), similar to findings of 0.39 in an FFQ validation study for Spanish pregnant women [22] and 0.46 in the study of pregnant women in rural China [9]. However, the ICCs of the two FFQs (0.22 to 0.71) in the present study were slightly lower than those in the study of Finnish pregnant women (0.44 to 0.91) [23]. 

We used FFQ2 to compare with the average of repeated 24 HR to evaluate the validity. The correlations of the frequencies of food groups between the two administrations (median 0.34, range 0.23–0.62) were slightly weaker than those found in other pregnant or non-pregnant populations. The Harvard Service FFQ (HSFFQ) used in low-income American Indian and Caucasian pregnant women was shown to have an average correlation coefficient of 0.35 between FFQ and 24 HR for energy-adjusted nutrient intakes [24]. The Shanghai Women’s Health Study (SWHS) reported that the correlation coefficients between its FFQ and 24 HR ranged from 0.41 to 0.66 [25]. The FFQ used among Norwegian pregnant women had average correlation coefficient of 0.48 for validity [26]. On the other hand, a validation study of a FFQ used among Malaysian pregnant women reported that the Spearman correlation coefficients for food groups intake between FFQ and 24 HR ranged from 0.13 (organ meats, onion and garlic) to 0.57 (malt drink) [27], which were a little lower than our findings. Overall, the median coefficient for validity in our study was within the acceptable to good range (*r* = 0.30–0.70), suggesting that the validity of the FFQ was reasonable [21]. In addition, we used cross-classification and the proportion of agreement to access the agreement between the two methods (FFQ vs. 24 HR). The mean proportion of the subjects classified into the same or adjacent quintile was 65.1%, while misclassified into distant quintile was 3.2%, which were similar or comparable to the levels of agreement in other validation studies in Chinese pregnant women [10,11]. 

The repeatability and validity of the present FFQ seems reasonable. It should be noted that all the studies mentioned above evaluated the validity and reproducibility of FFQ based on portion size or amount of nutrients intakes, whereas our study estimated based on frequencies of consumption. This difference in methodology may limit the direct comparability of our findings to those of previous studies.

In the present study, 210 participants completed all of the questionnaires and the 24 HR. The minimum sample size for validation studies is suggested at 100 participants [28], and Willett and Lenhart (1998) recommended a sample size of 100–200 participants for a validation study of dietary questionnaires [29]. Our study represented an appropriate sample size to assess the reliability of the FFQ. However, several limitations should be mentioned in the present study. First, there were only three repeated 24 HR to estimate the frequencies of dietary intake during the second and third trimesters. In addition, in the present study, the FFQ1 was completed by the pregnant women around 24 weeks and FFQ2 was completed around 32 weeks. The period between the two measurements (approximate 8-week interval) may be relatively short and the participants might have recalled their previous answers. This may contribute to the high correlations that we observed between the two FFQs. Actually, we considered starting the first FFQ at early pregnancy, which allows a longer period between FFQs. Unfortunately, pregnant women’s appetite fluctuations and nausea make the investigation at early pregnancy less accurate and feasible. Third, we used 24 HR as the reference method. Although a number of previous studies have also used 24 HR to assess the validity of FFQs, they were also susceptible to measurement error because of possible recall bias [29]. Fourth, there was a disparity in the time periods measured by the FFQ and 24 HR. The 24 HR collected foods intake data for three inconsecutive days during pregnancy, whereas the FFQ described the frequencies of foods consumed over a period of seven days. Therefore, the consumption frequencies of foods captured in both methods could differ. 

Another limitation of this FFQ was that it cannot be used to estimate the nutrients of foods intake among pregnant women, because data about dietary portion size were not collected in the FFQ. Dietary habits in Chinese population are complex. Food materials used in Chinese dishes are really diverse, especially in Cantonese areas. In addition, Chinese family members prefer to share their dishes on the table instead of piling food on a plate in Western areas. All of these make it difficult to estimate the accurate amount of each food item. Thus, we investigated the frequencies of food consumed, instead of portion sizes, and aimed to analyze the dietary pattern or food variety that could provide useful information on dietary habits. Regarding the quantitative data on dietary exposure, we intended to use blood samples to detect the levels of nutrients as internal exposure indicators for the pregnant women. 

In conclusion, our findings indicated that reproducibility and validity of the FFQ used in this study were acceptable. These findings supported the use of our FFQ to assess the usual consumption frequencies of foods among Chinese pregnant women in the BIGCS.

## Figures and Tables

**Table 1:** Characteristics of the validation study subjects, non-participating validation study women and Born in Guangzhou Cohort Study (BIGCS) cohort population.

	Validation Subjects	Non-Participants	Cohort Population	*p*_1_^†^	*p*_2_^‡^
*n*	210	210	10,165		
Age (years)	29.0 ± 3.2	29.8 ± 3.7	28.9 ± 3.4	0.174	0.674
Education level				0.584	<0.001 *
High school or below	10 (4.8)	11 (5.2)	1053 (11.2)		
College	44 (21.0)	48(22.9)	2371 (25.3)		
Undergraduate or above	156 (74.3)	151(71.9)	5947 (63.5)		
Monthly income (Yuan)				0.958	0.265
<1500	18 (8.9)	31 (15.4)	961 (10.5)		
1500–4500	62 (30.5)	48 (23.9)	2983 (32.5)		
4501–9000	87 (42.9)	79 (39.3)	3763 (41.0)		
≥9001	36 (17.7)	43 (21.4)	1468 (16.0)		
Pre-pregnancy BMI (kg/m^2^) ^#^				0.132	0.838
<18.5	46 (22.2)	38 (18.5)	2303 (24.8)		
18.5–23.9	146 (70.5)	143 (69.8)	6087 (65.7)		
≥24	15 (7.2)	24 (11.7)	879 (9.5)		
Parity				0.544	0.002 *
0	170 (81.7)	173 (84.0)	8309 (88.6)		
≥1	38 (18.3)	33 (16.0)	1071 (11.4)		

BIGCS, Born in Guangzhou Cohort Study; BMI, body mass index; GDM, gestational diabetes mellitus; **^†^** *p*-value of the differences between the validation study subjects and non-participating validation study women; ^‡^ *p*-value of the differences between the validation study subjects and cohort population; * Significantly different from the BIGCS cohort population, *p* < 0.01 (*t-*tests or Kruskal-Wallis Test). Values are reported as mean ± standard deviation or *n* (%); ^#^ Pre-pregnancy body mass index (BMI) (kg/m^2^) was divided into three groups according to the Guidelines for Prevention and Control of Overweight and Obesity in Chinese Adults [20].

**Table 2:** Comparison of the median and mean daily food consumption frequencies between the first and second food frequency questionnaires (FFQ1 and FFQ2) and correlation coefficients of FFQ1 and FFQ2.

	FFQ1		FFQ2		FFQ2/FFQ1 × 100	*p*-Value *	Spearman Correlation Coefficient	ICC
	Median (P25, P75) ^‡^	Mean (SD)	Median (P25, P75) ^‡^	Mean (SD)	Median			
Red and processed meats	1.43 (0.86, 2.00)	0.76 (1.43)	1.43 (1.00, 2.00)	1.58 (0.80)	100	0.12	0.40	0.47
Poultry	0.43 (0.29, 0.57)	0.31 (0.43)	0.43 (2.00, 4.00)	3.00 (1.42)	100	0.03	0.47	0.42
Eggs	0.86 (0.57, 1.00)	0.40 (0.86)	0.86 (0.43, 1.00)	0.80 (0.39)	100	0.03	0.45	0.43
Fish	0.43 (0.29, 0.57)	0.35 (0.43)	0.43 (0.29, 0.71)	0.49 (0.33)	100	0.37	0.55	0.45
Sea food	0.14 (0.00, 0.29)	0.17 (0.14)	0.14 (0.00, 0.29)	0.15 (0.18)	100	0.46	0.45	0.43
Soybean	0.57 (0.29, 1.00)	0.60 (0.57)	0.57 (0.39, 1.00)	0.74 (0.51)	100	0.83	0.57	0.52
Other legumes	0.14 (0.00, 0.29)	0.20 (0.14)	0.14 (0.00, 0.29)	0.19 (0.19)	100	0.38	0.35	0.37
Leafy vegetables	1.86 (1.25, 2.29)	1.06 (1.86)	1.86 (1.29, 2.43)	1.93 (0.89)	100	0.66	0.42	0.43
Root vegetables	0.43 (0.29, 0.71)	0.37 (0.43)	0.43 (0.29, 0.71)	0.56 (0.34)	100	0.82	0.41	0.4
Melon vegetables	0.57 (0.29, 0.71)	0.44 (0.43)	0.43 (0.14, 0.57)	0.44 (0.34)	75	<0.001	0.46	0.48
Mushrooms and fungus	0.14 (0.00, 0.29)	0.19 (0.14)	0.14 (0.00, 0.29)	0.20 (0.20)	100	0.71	0.38	0.31
Seaweed	0.00 (0.00, 0.14)	0.12 (0.00)	0.00 (0.00, 0.14)	0.10 (0.12)	0	15	0.33	0.33
Pickled vegetables	0.00 (0.00, 0.14)	0.15 (0.00)	0.00 (0.00, 0.00)	0.05 (0.12)	0	0.03	0.37	0.22
Fruits	1.14 (0.86, 1.61)	0.61 (1.14)	1.14 (0.86, 1.57)	1.25 (0.64)	100	0.33	0.48	0.33
Nuts	0.57 (0.29, 1.00)	0.45 (0.57)	0.57 (0.29, 1.00)	0.59 (0.43)	100	0.004	0.48	0.47
Milk	1.00 (0.57, 1.14)	0.58 (0.86)	0.86 (0.43, 1.14)	0.90 (0.60)	86	0.15	0.46	0.39
Cereals and grains	3.00 (2.57, 3.43)	0.66 (3.00)	3.00 (2.43, 3.29)	2.89 (0.78)	100	0.06	0.37	0.39
Yogurt	0.14 (0.00, 0.43)	0.31 (0.14)	0.14 (0.00, 0.43)	0.25 (0.31)	100	0.25	0.38	0.39
Soup	0.50 (0.21, 0.79)	0.33 (0.50)	0.50 (0.21, 0.84)	0.56 (0.34)	100	0.4	0.71	0.71
Average							0.45	0.42

* Differences were tested using Wilcoxon signed rank tests. ^‡^ 25th, 75th percentiles. ICC: Intraclass correlation coefficients.

**Table 3:** Comparison of the median daily food consumption frequencies between the second food frequency questionnaire (FFQ2) and three 24 HR and Spearman correlation coefficients of FFQ2 and average 24 HR.

Food Group	Average of Three 24 HR	FFQ2	*p*-Value *	FFQ2/24 HR×100	Spearman Correlation Coefficient
	Median (Frequency)	Median (Frequency)		(Median Frequency)	
Red meats	1.67	1.43	0.02	117	0.25
Poultry	0.67	0.43	<0.001	156	0.38
Eggs	0.67	0.86	0.27	78	0.35
Fish	0.50	0.43	0.01	117	0.43
Seafood	0.00	0.14	0.003	-	0.47
Legumes	0.33	0.57	<0.001	58	0.27
Other legumes	0.00	0.14	<0.001	-	0.29
Leafy vegetables	1.67	1.86	0.002	90	0.23
Root vegetables	0.33	0.43	<0.001	78	0.35
Melon vegetables	0.33	0.43	0.78	78	0.31
Mushrooms and fungus	0.33	0.14	<0.001	233	0.29
Seaweed	0.00	0.00	<0.001	-	0.25
Pickled vegetables	0.00	0.00	0.02	-	0.23
Fruits	1.50	1.14	<0.001	131	0.40
Nuts	0.33	0.57	<0.001	58	0.37
Milk	0.67	0.86	0.012	78	0.62
Cereals and grains	3.00	3.00	0.04	100	0.32
Yogurt	0.00	0.14	<0.001	-	0.41
Soup	0.67	0.50	<0.001	133	0.30
Average					0.34

* Differences in frequencies were tested using Wilcoxon signed rank tests.

**Table 4:** Cross-classification of intakes of food groups based on the average of two FFQs and the average of three 24 HR.

Food Groups	Same Quintile (%)	Same or Adjacent Quintile (%)	Distant Quintile (%)
Red and processed meats	21	65.2	4.8
Poultry	32.9	66.2	3.3
Eggs	30	69.5	3.8
Fish	25.2	68.1	1
Seafood	29	71.4	1
Legumes	23.8	57.6	2.9
Other legumes	25.2	58.6	3.8
Leafy vegetables	23.8	57.1	3.3
Root vegetables	28.1	61.9	1.9
Melon vegetables	30.5	67.1	5.7
Mushrooms and fungus	23.8	65.7	4.8
Seaweed	24.3	60	3.3
Pickled vegetables	25.2	57.6	3.8
Fruits	29	69	2.9
Nuts	31	68.6	1.4
Milk	43.8	79	1.4
Cereals and grains	28.1	63.3	3.3
Yogurt	28.6	63.3	3.3
Soup	30.5	67.6	4.8

## Supplementary Materials

The following are available online at http://www.mdpi.com/2072-6643/8/8/454/s1, Figure S1: Bland-Altman plots of food groups for representing the differences between the mean consumption frequencies from the two food frequency questionnaires (FFQ1 and FFQ2), Table S1: List of food items included in the 19 main food groups.

## References

[1] Schoenaker D.A., Soedamah-Muthu S.S., Mishra G.D. (2014). The association between dietary factors and gestational hypertension and pre-eclampsia: A systematic review and meta-analysis of observational studies. BMC Med..

[2] Saunders L., Guldner L., Costet N., Kadhel P., Rouget F., Monfort C., Thome J.P., Multigner L., Cordier S. (2014). Effect of a mediterranean diet during pregnancy on fetal growth and preterm delivery: Results from a french caribbean mother-child cohort study (timoun). Paediatr. Perinat. Epidemiol..

[3] Acuna-Muga J., Ureta-Velasco N., de la Cruz-Bertolo J., Ballesteros-Lopez R., Sanchez-Martinez R., Miranda-Casabona E., Miguel-Trigoso A., Garcia-San Jose L., Pallas-Alonso C. (2014). Volume of milk obtained in relation to location and circumstances of expression in mothers of very low birth weight infants. J. Hum. Lact..

[4] Mohammadbeigi A., Farhadifar F., Soufi Zadeh N., Mohammadsalehi N., Rezaiee M., Aghaei M. (2013). Fetal macrosomia: Risk factors, maternal, and perinatal outcome. Ann. Med. Health Sci. Res..

[5] Born in Guangzhou Cohort Study (Bigcs). https://clinicaltrials.Gov./ct2/show/nct02526901.

[6] Rifas-Shiman S.L., Rich-Edwards J.W., Willett W.C., Kleinman K.P., Oken E., Gillman M.W. (2006). Changes in dietary intake from the first to the second trimester of pregnancy. Paediatr. Perinat. Epidemiol..

[7] Durnin J.V. (1991). Energy requirements of pregnancy. Diabetes.

[8] Butte N.F., Wong W.W., Treuth M.S., Ellis K.J., O’Brian Smith E. (2004). Energy requirements during pregnancy based on total energy expenditure and energy deposition. Am. J. Clin. Nutr..

[9] Cheng Y., Yan H., Dibley M.J., Shen Y., Li Q., Zeng L. (2008). Validity and reproducibility of a semi-quantitative food frequency questionnaire for use among pregnant women in rural China. Asia Pac. J. Clin. Nutr..

[10] Li M., Halldorsson T.I., Bjerregaard A.A., Che Y., Mao Y., Hu W., Wang Y., Zhou W., Olsen S.F., Strom M. (2014). Relative validity and reproducibility of a food frequency questionnaire used in pregnant women from a rural area of China. Acta Obstet. Gynecol. Scand..

[11] Zhang H., Qiu X., Zhong C., Zhang K., Xiao M., Yi N., Xiong G., Wang J., Yao J., Hao L. (2015). Reproducibility and relative validity of a semi-quantitative food frequency questionnaire for chinese pregnant women. Nutr. J..

[12] Zhang C.X., Ho S.C. (2009). Validity and reproducibility of a food frequency questionnaire among Chinese women in Guangdong province. Asia Pac. J. Clin. Nutr..

[13] He J.R., Yuan M.Y., Chen N.N., Lu J.H., Hu C.Y., Mai W.B., Zhang R.F., Pan Y.H., Qiu L., Wu Y.F. (2015). Maternal dietary patterns and gestational diabetes mellitus: A large prospective cohort study in China. Br. J. Nutr..

[14] Lu M.S., Chen Q.Z., He J.R., Wei X.L., Lu J.H., Li S.H., Wen X.X., Chan F.F., Chen N.N., Qiu L. (2016). Maternal dietary patterns and fetal growth: A large prospective cohort study in China. Nutrients.

[15] Chinese Nutrition Society (2009). Dietary Guidelines for Chinese Residents.

[16] Yang Y., Wang G., Pan X. (2009). China Food Composition.

[17] Marques-Vidal P., Ross A., Wynn E., Rezzi S., Paccaud F., Decarli B. (2011). Reproducibility and relative validity of a food-frequency questionnaire for french-speaking swiss adults. Food Nutr. Res..

[18] Wong J.E., Parnell W.R., Black K.E., Skidmore P.M. (2012). Reliability and relative validity of a food frequency questionnaire to assess food group intakes in New Zealand adolescents. Nutr. J..

[19] Bland J.M., Altman D.G. (1986). Statistical methods for assessing agreement between two methods of clinical measurement. Lancet.

[20] Chen C., Lu F.C. (2004). The guidelines for prevention and control of overweight and obesity in Chinese adults. Biomed. Environ. Sci..

[21] Willett W. (2009). Foreword. The validity of dietary assessment methods for use in epidemiologic studies. Br. J. Nutr..

[22] Vioque J., Navarrete-Munoz E.M., Gimenez-Monzo D., Garcia-de-la-Hera M., Granado F., Young I.S., Ramon R., Ballester F., Murcia M., Rebagliato M. (2013). Reproducibility and validity of a food frequency questionnaire among pregnant women in a mediterranean area. Nutr. J..

[23] Erkkola M., Karppinen M., Javanainen J., Rasanen L., Knip M., Virtanen S.M. (2001). Validity and reproducibility of a food frequency questionnaire for pregnant Finnish women. Am. J. Epidemiol..

[24] Stuff J.E., Goh E.T., Barrera S.L., Bondy M.L., Forman M.R. (2009). N-nitroso compounds: Assessing agreement between food frequency questionnaires and 7-day food records. J. Am. Diet. Assoc..

[25] Shu X.O., Yang G., Jin F., Liu D., Kushi L., Wen W., Gao Y.T., Zheng W. (2004). Validity and reproducibility of the food frequency questionnaire used in the shanghai women’s health study. Eur. J. Clin. Nutr..

[26] Brantsaeter A.L., Haugen M., Alexander J., Meltzer H.M. (2008). Validity of a new food frequency questionnaire for pregnant women in the Norwegian mother and child cohort study (moba). Matern. Child Nutr..

[27] Loy S.L., Marhazlina M., Nor A.Y., Hamid J.J. (2011). Development, validity and reproducibility of a food frequency questionnaire in pregnancy for the universiti sains Malaysia birth cohort study. Malays. J. Nutr..

[28] Cade J., Thompson R., Burley V., Warm D. (2002). Development, validation and utilisation of food-frequency questionnaires-a review. Public Health Nutr..

[29] Willett W., Lenart E. (1998). Reproduciblity and validity of food-frequency questionnaires. Nutritional Epidemiology.

