# Supplementary material for: Validity and Reproducibility of a Dietary Questionnaire for Consumption Frequencies of Foods during Pregnancy in the Born in Guangzhou Cohort Study (BIGCS)
Source: Nutrients. 2016 Jul 28;8(8):454. doi: 10.3390/nu8080454 (PMC4997369; doi:10.3390/nu8080454)
Supplement: Supplementary file 1 [file nutrients-08-00454-s001.doc]

Supplementary Materials: Validity and Reproducibility of a Dietary Questionnaire for Consumption Frequencies of Foods during Pregnancy in the Born in Guangzhou Cohort Study (BIGCS)

**Ming-Yang Yuan, Jian-Rong He, Nian-Nian Chen, Jin-Hua Lu, Song-Ying Shen, Wan-Qing Xiao, Fang Hu, Hui-Yun Xiao, Yan-Yan Wu, Xiao-Yan Xia, Yu Liu, Lan Qiu, Ying-Fang Wu, Cui-Yue Hu, Hui-Min Xia and Xiu Qiu**

|  |  |
| --- | --- |
| (**a**) | (**b**) |
|  |  |
| (**c**) | (**d**) |
|  |  |
| (**e**) | (**f**) |
|  |  |
| (**g**) | (**h**) |
|  |  |
| (**i**) | (**j**) |
|  |  |
| (**k**) | (**l**) |
|  |  |
| (**m**) | (**n**) |
|  |  |
| (**o**) | (**p**) |
|  |  |
| (**q**) | (**r**) |
|  |  |
| (**s**) |  |

**Figure S1.** Bland-Altman plots of food groups for representing the differences between the mean consumption frequencies from the two food frequency questionnaires (FFQ1 and FFQ2). Solid lines represent mean difference, and dashed lines show lower and upper 95% limits of agreement (mean ± 1.96 SD). (**a**) Bland-Altman plots for red and processed meats (**b**) Bland-Altman plots for poultry (**c**) Bland-Altman plots for eggs (**d**) Bland-Altman plots for fish (**e**) Bland-Altman plots for sea food (**f**) Bland-Altman plots for soybean (**g**) Bland-Altman plots for other legumes (**h**) Bland-Altman plots for leafy vegetables (**i**) Bland-Altman plots for root vegetables (**j**) Bland-Altman plots for melon vegetables (**k**) Bland-Altman plots for mushrooms and fungus (**l**) Bland-Altman plots for seaweed (**m**) Bland-Altman plots for pickled vegetables (**n**) Bland-Altman plots for fruits (**o**) Bland-Altman plots for nuts (**p**) Bland-Altman plots for milk (**q**) Bland-Altman plots for cereals and grains (**r**) Bland-Altman plots for yogurt (**s**) Bland-Altman plots for soup.

**Table S1.** List of food items included in the 19 main food groups.

| **Foods or Food Groups** | **Food Items** |
| --- | --- |
| Red and processed meats | Pork; beef; lamb or mutton; processed meat (including sausage, salami, and luncheon meat); liver; blood products; brains; other offals |
| Poultry | Chicken, duck, goose |
| Eggs | Fresh eggs (egg white, egg yolk and whole egg); preserved eggs |
| Fish | Marine fish; river fish |
| Seafood | Shrimp, crab; scallops, clams, oysters; other shellfish |
| Soybean | Soybeans; soy milk ; other soybean products |
| Other legumes | Other beans; peas |
| Leafy vegetables | Dark green leafy vegetables; other leafy vegetables; broccoli; other cruciferous vegetable |
| Root vegetables | Carrot; radish; other starchy tubers |
| Melon vegetables | Pumpkin, tomato, eggplant; bitter melon, cucumber, sponge gourd, wax gourd |
| Mushrooms and fungus | Mushrooms; fungus |
| Seaweed | seaweed, laver, kelp |
| Pickled vegetables | Pickled vegetables |
| Fruits | Apple and other low sugar fruits; banana and other medium sugar fruits; mango and other high sugar fruits |
| Nuts and seeds | Nuts, walnuts, hazelnuts, almonds, pistachios, pine nuts, sunflower seed, cashew nuts, and sunflower seed; chestnuts, lotus seed; other nuts and seeds |
| Milk | Fresh full fat milk; full fat milk powder; low fat fluid milk; low fat milk powder; other milk beverage |
| Cereals and grains | White rice; porridge; oats; corns; noodles; pasta; pizza; dumplings, buns; bread; cake; biscuits; sweet potato, potato; other cereals or grains |
| Yogurt | Yogurt |
| Soup | Soup |
